# Spatio-Temporal Distribution of Vector-Host Contact (VHC) Ratios and Ecological Niche Modeling of the West Nile Virus Mosquito Vector, *Culex quinquefasciatus,* in the City of New Orleans, LA, USA

**DOI:** 10.3390/ijerph14080892

**Published:** 2017-08-08

**Authors:** Mohamed F. Sallam, Sarah R. Michaels, Claudia Riegel, Roberto M. Pereira, Wayne Zipperer, B. Graeme Lockaby, Philip G. Koehler

**Affiliations:** 1 Department/School Entomology and Nematology Department, University of Florida, Gainesville, FL 32611, USA; rpereira@ufl.edu (R.M.P.); pgk@ufl.edu (P.G.K.); 2 College of Science, Ain Shams University, Cairo 11566, Egypt; 3 City of New Orleans Mosquito & Termite Control Board, 2100 Leon C. Simon, New Orleans, LA 70122, USA; srmichaels@nola.gov (S.R.M.); criegel@nola.gov (C.R.); 4 Research Forester, USDA Forest Service, P.O. Box 110806, Gainesville, FL 32611-0806, USA; wzipperer@fs.fed.us; 5 Center for Environmental Studies at the Urban-Rural Interface (CESURI), School of Forestry & Wildlife Sciences, Auburn University, Auburn, AL 36849, USA; lockabg@auburn.edu

**Keywords:** West Nile virus, *Culex quinquefasciatus*, habitat suitability, New Orleans, distribution risk

## Abstract

The consistent sporadic transmission of West Nile Virus (WNV) in the city of New Orleans justifies the need for distribution risk maps highlighting human risk of mosquito bites. We modeled the influence of biophysical and socioeconomic metrics on the spatio-temporal distributions of presence/vector-host contact (VHC) ratios of WNV vector, *Culex quinquefasciatus*, within their flight range*.* Biophysical and socioeconomic data were extracted within 5-km buffer radii around sampling localities of gravid female *Culex quinquefasciatus*. The spatio-temporal correlations between VHC data and 33 variables, including climate, land use-land cover (LULC), socioeconomic, and land surface terrain were analyzed using stepwise linear regression models (RM). Using MaxEnt, we developed a distribution model using the correlated predicting variables. Only 12 factors showed significant correlations with spatial distribution of VHC ratios (*R*^2^ = 81.62, *p* < 0.01). Non-forested wetland (NFWL), tree density (TD) and residential-urban (RU) settings demonstrated the strongest relationship. The VHC ratios showed monthly environmental resilience in terms of number and type of influential factors. The highest prediction power of RU and other urban and built up land (OUBL), was demonstrated during May–August. This association was positively correlated with the onset of the mosquito WNV infection rate during June. These findings were confirmed by the Jackknife analysis in MaxEnt and independently collected field validation points. The spatial and temporal correlations of VHC ratios and their response to the predicting variables are discussed.

## 1. Introduction

West Nile virus (WNV) was first reported in 1999 in New York City, NY, USA. By 2000 the disease has spread throughout the northeastern USA [1,2,3]. The virus reached Louisiana in the fall of 2001, when a dead crow in Jefferson Parish was identified as being infected with WNV [4]. By 2003, WNV infections occurred in 60 of the 64 Louisiana’s parishes. In the New Orleans metropolitan areas (Orleans and Jefferson Parishes) focal transmission activity occurs principally during mid-July [4].

*Culex quinquefasciatus* Say, as the main vector, with *Cx. salinarius* Coquillett possibly acting as a secondary vector, were incriminated in the WNV outbreak in southern Louisiana during 2002 [5,6,7]. The former mosquito species, with a feeding preference for mammals, was responsible for enzootic/epidemic transmission, especially in urban and sub-urban settings [7,8,9,10,11,12,13,14]. The primary mosquito vector showed biological and ecological resilience in space and time based on the available environmental resources. This resilience may influence the spatio-temporal distribution of the WNV vector, which may or may not bring them to the vicinity of both reservoir host(s) and human populations. Eventually this will affect the amplification and transmission cycles of WNV in areas under risk.

In New Orleans, the confluence of availability of competent mosquito vector(s), susceptible reservoir host(s), suitable natural systems and climate for both mosquitoes and host(s) enabled the autochthonous transmission of WNV with hundreds of human cases and major mortality of wild native and exotic birds [2,4]. Nonetheless, the transmission dynamics of WNV in terms of space and time in relationship to the biology, ecology of mosquito vector(s), and their biophysical systems remains unclear. In fact, the distribution, blood-feeding preference, flight range and vectorial capacity of mosquito vectors are very critical inputs for predicting the transmission cycle of this disease.

Furthermore, mosquito vectors often shift their feeding preference seasonally or spatially, depending on the availability of the blood meal source. For example, *Cx. quinquefasciatus* showed an opportunistic preference for blood meal. In peninsular Florida, it is responsible for an epizootic cycle and sustaining the virus circulation within reservoir host bird(s) [15,16]. However, it has been incriminated with the enzootic/epidemic transmission cycle of WNV in urban and sub-urban areas in Louisiana due to feeding preference to humans and other mammals [4,6,7,17,18,19].

Currently, most species distribution models for mosquitoes are based on hydrological and meteorological data [15,16,20,21,22]. Some models include socio-environmental predictors in terms of vegetation or urban and sub-urban areas [23]. With respect to WNV, models have used either used data points of WNV cases and mosquito vectors instead of the flight range of the mosquito vectors around their hosts or predicted the distribution risk of WNV on regional scale. Prediction models for WNV and Zika virus (ZIKV) transmission potential were generated for their mosquito vectors in regard to their flight range around recorded positive cases highlighting their response to surrounding biophysical systems such as climate and non-climate factors [24,25]. Although previous models are useful, their findings did not adequately account for the comprehensive response of vector-host contact (VHC) ratios to climate and non-climate variables such as land use-land cover (LULC) and Digital Elevation Models (DEM) and the overall influence on arbovirus transmission potential [26,27]. Mosquito density reflect neither the likelihood of biting risk, which is caused by mosquito vector, nor the transmission potential as a function of biting rate. The VHC explains the ratio between collected mosquito density and human population census, which reflects areas under risk of increased biting rate by mosquito vector.

The lack of available vaccines for WNV and consistent development of insecticide resistance for mosquito vector populations jeopardize public health in affected areas. Additionally, the focal and sporadic locally-transmitted cases justify the necessity to generate prediction models that identifies areas under risk of infective biting rates in order to target during surveillance and control activities. In our model, we evaluated the spatio-temporal distribution of VHC ratios in response to: (i) future climate scenarios during 2011–2030, (ii) LULC, (iii) socioeconomic, and (iv) DEM systems. Our correlative models were generated within the flight range of WNV vector in the city of New Orleans, LA (NOLA). The spatio-temporal VHC ratios were estimated utilizing data records on female gravid mosquito and human population census per block during 2015. The spatio-temporal resilience of VHC ratios to their predicted biophysical systems was characterized. This allowed developing prediction risk maps for the WNV vector presence using the Maximum Entropy (MaxEnt) tool, emphasizing the human population under risk of infective mosquito bites*.* Since the local economy in NOLA is primarily driven by tourism, management of arbovirus diseases has a significant economic implications. Arbovirus transmission has the potential to jeopardize the tourism industry, making NOLA surveillance and control programs very important to the economic and ecological health of the city.

## 2. Materials and Methods

### 2.1. Study Area

New Orleans, Louisiana (NOLA) lies on the Mississippi River, near the Gulf of Mexico with a total area of 1084 km^2^ inhabited by almost 1,262,888 people, with an average density of 1165/km^2^. NOLA is sub-tropical with an annual high temperature of 25 °C, an annual low of 16.8 °C, and average annual precipitation of 162.3 cm. Average highest precipitation occurs in July (17.9 cm).

### 2.2. Data Layers

#### 2.2.1. Mosquito Sampling and Socioeconomic Data

Density of wild-collected female gravid *Cx. quinquefasciatus* was estimated using Center for Disease Control (CDC) gravid traps (John W. Hock Company, Gainesville, FL, USA) at 37 permanent locations in the City of New Orleans. Traps were placed outdoors at the beginning of April through the last week of December during 2015. Traps were setup on the ground and operated for 18–20 h using a 12-v battery. Mosquito collections were transported to the laboratory facility at The NOLA Mosquito Control Board for further identification to the species level using the taxonomic keys of Darsie and Ward [28].

Because the density of collected mosquitoes reflects neither the risk of vector contact with host nor disease transmission rates, the vector-host contact (VHC) ratios were calculated within 5-km buffer radii around the sampling localities [25,27,29]. These buffer radii reflect the foraging activity of both ovipositing and newly emerged host seeking mosquitoes from their breeding sites. Moreover, these buffer radii demonstrate human populations under risk of bites of host seeking mosquito and disease transmission. Although the sampled mosquitoes in the current study were gravid and not host seeking, their density gives insight regarding the wild mosquito populations within 5-km radii around sampling sites.

The VHC ratios were estimated utilizing spatial and temporal density of WNV vector and human population census within flight buffer radii (~5-km) of this mosquito vector. For the spatial analysis, density of the mosquito vector (total number of mosquito vector/season/5-km) was estimated for each sampling site, whereas in the temporal analysis, density was estimated on a monthly basis. Data on human population census/housing block was imported from the NOLA census records of 2015 (data.nola.gov), and clipped within the buffer radii around vector sampling sites. Extraction of both mosquito density and human population census were conducted using the Arc toolbox in ArcGIS ver. 10.1 (Esri, Redlands, CA, USA). Accordingly, the spatio-temporal fluctuation of VHC ratios were estimated in response to their predicted biophysical systems within the flight buffer radii.

#### 2.2.2. Bioclimatic Data

In order to demonstrate the spatio-temporal fluctuation of VHC ratios in response to future climate scenarios in NOLA, we utilized climate data from 2011–2030 (Table 1). Utilization of future climate scenarios not only highlights the sampling time frame of WNV vector during 2015, but also projects the future distribution of VHC ratios. Bioclimatic data layers were obtained from the General Circulation Models (GCMs) for the optimistic IPCC Special Range of Emission Scenarios (SRES A1B). The A1B represents a medium and balanced scenario for the emission rate produced by Green House Gases (GHG). This scenario was imported from the data base at Centro International de Agricultura Tropical (CIAT) (http://www.ccafs-climate.org/data/) [30]. We also used the data based on the global circulation model CSIRO-Mk3.5.0. For projections, a spatial resolution of 30 arc sec (~1 km) was applied. The layers were clipped to match dimensions of NOLA and saved as ASCII grids using Model Builder in ArcGIS ver. 10.1 (Esri, Redlands, CA, USA).

The DEMs representing slope, aspect ratio, curvature, and hill shade were highlighted in previous studies to predict land geomorphology, temporary water accumulation and probable breeding sites for mosquitoes [25,32,33,34,35]. Accordingly, these land surface indicators were generated from a 30 arc-seconds DEM to be included in our investigation. Land geomorphology and temporary water accumulations within 1 km were not highlighted in our study, because of two reasons: (i) resolution of all data layers were resampled to match worldclim data (~1 km), (ii) *Cx quinquefasciatus* has been reported to fly for longer distances within ~5 km seeking suitable host(s) and breeding sites.

#### 2.2.3. Land Use-Land Cover Data (LULC)

Since mosquitoes depend on the human population as a source of blood meal [36], an urban areas layer was included as a predictor (Table 1). Urban areas were categorized into three classes to represent degree of urbanization: (1) residential and urban, in which housing predominates in two different forms; (2) industrial and commercial services, which represents industrial settings and fewer housing structures; and (3) other urban and built-up land with the least housing and human populations.

Vegetation at the site level (data from USDA Forest Service and Tulane University), representing resting places and sugar meal sources for adult mosquitoes, was included in our model [37,38,39]. Additionally, vegetation reflected habitat quality for nesting birds [40,41], which are reservoir hosts of WNV. Therefore, four classes of vegetation types were extracted from the USGS and literature [31] to build up our model: (1) non-forested wetland, (2) forest wetland, (3) deciduous forestland, and (4) tree density.

Streams, canals, lakes, reservoirs and estuaries of different sizes (> and ≤1 km) were also included to represent permanent water bodies, as possible breeding sites for *Cx*. *quinquefasciatus*. All LULC data layers were imported from a US Geological Survey (USGS) data set. These data were built during the 1970s and 1980s and updated and released during 2007 [42]. For comparison and confirmation of the USGS data, areas of LULC from zoning district data layers were imported from the City of New Orleans Enterprise GIS Database during 2016.

**Figure 1:**
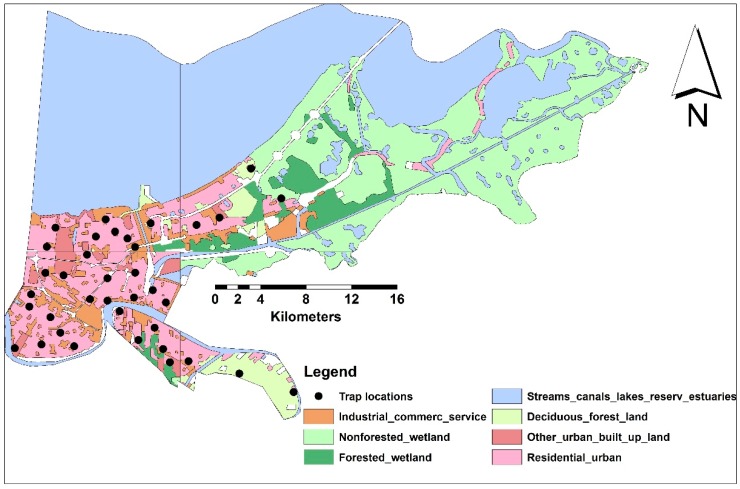
The seven LULC classes and sampling sites in NOLA.

Although sampling sites were randomly selected to represent all LULC classes within the buffer radii, urban areas were extensively highlighted in our study to predict human population under risk of increased biting rate (Figure 1, Table 2). Additionally, the area percentages of each LULC class to the total sampled areas within 5-km buffer radii were estimated (Table 2).

### 2.3. Variables Selection

**Figure 2:**
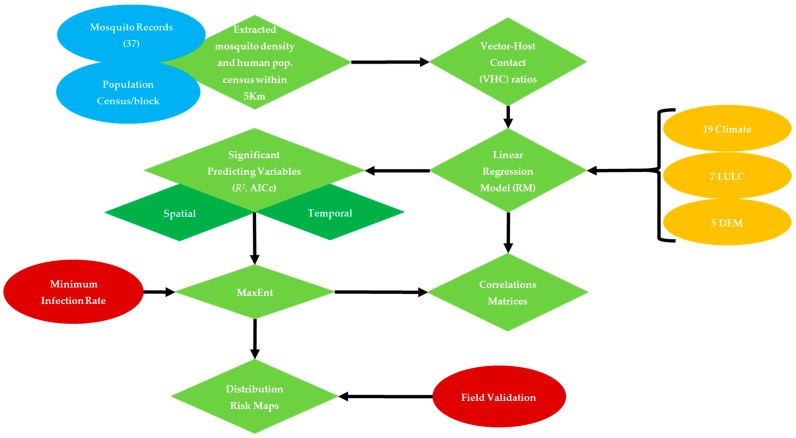
West Nile Virus transmission model, and expected outcomes in response to proposed predicting variables.

A total of 33 bioclimatic, LULC, socioeconomic and DEM data layers (Table 1) were clipped to NOLA and extracted within each 5-km buffer radii around mosquito sampling sites in preparation for collinearity analysis to: (i) reduce redundancy between influential factors, and (ii) select the significant explanatory variables to be included in MaxEnt (Figure 2) [25,27]. In this regard, a stepwise linear regression model (RM) was carried out for three purposes: (i) to test the spatial dependency of vector-host contact ratios on their predicting variables within the flight range radii of this mosquito vector around their sampling sites, (ii) to characterize monthly resilience of *Cx. quinquefasciatus* to their predicting variables, (iii) to overcome redundancy and exclude the linearly correlated variables. This analysis was carried out using JMP pro statistical package ver. 10.0.0 [43]. The minimum corrected Akaik Information Criterion (AICc) and *R*^2^ values were used to select the significant predicting variables (*p* < 0.05) [25,44,45,46,47].

### 2.4. Habitat Suitability Modeling of WNV Vector

The maximum likelihood of habitat suitability for *Cx. quinquefasciatus* was modeled using MaxEnt software v. 3.3 (http://www.cs.princeton.edu/~schapire/maxent/, New York, NY, USA), [48,49,50]. This analysis uses the occurrence records of mosquito vectors in association with the selected predicting variables from RM to generate suitability risk maps. Accordingly, the Jackknife test was used to evaluate the permutation importance of independent variables in our model. The generated risk probability was categorized into five classes using the natural area breaks in ArcGIS utilizing WNV minimum infection rate (0.8/1000) recorded by Godsey et al. (2005) [5]: very low (0–0.2), low (>0.2–0.4), medium (>0.4–0.6), high (>0.6–0.8), and very high (>0.8).

The 37 collection sites were used as spatio-temporal replicates for the mosquito vector distribution. These records were randomly partitioned for model evaluation into two subsamples: 75% of the records were used for training and building the model, and 25% of the records were used for testing the model’s accuracy. The duplicate records of WNV mosquito vectors within ~1-km of the same cell size were excluded [51]. During data training, a matrix of spatial correlations between sampling points and their associated predicting variables were created. Accordingly, the habitat suitability maps were created for sampled and unsampled areas based on the habitat similarity between sampled and unsampled regions.

Five replicate runs were assigned in running the model to generate the average, maximum, minimum and median of the distribution range of mosquito vectors. Prediction models were evaluated using the cross-validation method, by systematically removing each data point from our training data set and predicting the removed point based on the remaining data points. Two thresholds have been used to examine the performance accuracy of our model [49]: (i) the extrinsic omission was evaluated at a fixed threshold (10 percentile training presence), and (ii) the area under the curve (AUC) of the receiver operating characteristics (ROC).

To increase the potential of the habitat suitability model and maximize the sampling effort of extracted mosquito vectors, a separate ASCII file generated from the 5-km buffer zones was included and weighted to the corresponding VHC ratios [25,52]. Accordingly, the habitat suitability of the gravid mosquito vector will be predicted in regard to their flight range around human hosts, breeding habitats and in response to their predicting variables.

Therefore, the generated risk map reflects the association between *Cx. quinquefasciatus*, available avian or human blood meal, and water habitats. The MaxEnt evaluates different correlations between the presence records of extracted mosquito vector data and their predicting variables within the sampled areas utilizing logistic regression analysis. In our study, we weighted the presence records to VHC ratios 5-km around sampling localities and attached these as ASCII bias files. Since the habitat suitability for WNV transmission generated by MaxEnt was produced at the threshold of WNV minimum infection rate, our risk maps highlighted human populations under risk of infective mosquito bites.

### 2.5. Model Validation

To evaluate the generated risk probability maps, a total of 18 independent field validation points were sampled biweekly. The validation points were randomly selected to represent areas with high mosquito and human population density. Female mosquitoes (~4–100) sampled from each locality were pooled according to their date of collection in order to be used for WNV testing. The sampling sites were identified as WNV positive as long as one pool was reported positive from the same locality during the season

## 3. Results

### 3.1. Variables Selection

#### 3.1.1. Spatial Analysis

The vector-host contact ratios showed variation in their response to the 32 variables used in RM. The increase in VHC ratios showed significant correlations with 12 predicting variables (AICc = 505.01, *R*^2^ = 81.62, *p* < 0.01) (Table 3). The LULC related variables were found to be the key predictors, especially non-forested wetland (NFWL) (*r*_(13)_ = 1.91, AICc = 505.01, *R*^2^ = 81.62, *p* < 0.01). This land cover type alongside with tree density (TD) positively correlated with the vector-host contact ratios. However, the increase in residential and urban settings shared a reduced negative influence on the spatial distribution of WNV mosquito vector (Table 4).

Three of the predictors were related to seasonal precipitation and temperature variables namely: precipitation of wettest month (Bio13), and precipitation of driest (Bio17) and warmest quarters (Bio18). The coefficient estimates in RM demonstrated a negative association between WNV mosquito vector and Bio17 and Bio18. Additionally, six temperature related variables correlated with VHC ratios namely: mean annual temperature (Bio1), mean diurnal range (Bio2), temperature seasonality (Bio4), minimum temperature of coldest month (Bio6), temperature annual range (Bio7), and mean temperature of coldest quarter (Bio11). Temperature related variables had varied correlations with VHC ratios. Although the increased contact ratios showed a positive association with warm sampling localities (Bio1, Bio4, and Bio11), Bio2, Bio6 and Bio7 showed negative correlations (Table 4).

#### 3.1.2. Temporal Analysis

**Figure 3:**
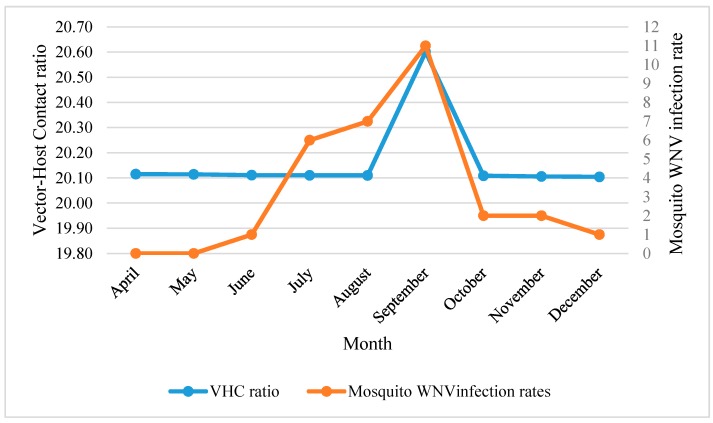
Monthly mosquito WNV infection rate in correlation with vector-host contact ratios in NOLA.

The temporal distribution of WNV mosquito vector showed ecological resilience in terms of month-to-month response to their predicting variables. This resilience was demonstrated as the temporal changes of predicting variables and did not affect monthly vector-host contact ratios (20.16 ± 0.02) (Figure 3).

Generally, temperature related variables were the key factors in predicting monthly distribution of WNV mosquito vector (Table 5). These variables were manifested as: mean temperature of coldest (Bio11), wettest (Bio8) and driest (Bio9) quarters, mean annual temperature (Bio1), and mean diurnal range (Bio2). The distribution of WNV mosquito density was shown to be positively associated with the increase of all temperature parameters during sampling time. The increase in density of WNV mosquito vector during April–September was found to be positively associated with Bio11. This correlation was demonstrated as maximum prediction probability of vector-host contact ratios during September (*r*_(4)_ = 0.96, AICc = 88.26, *R*^2^ = 58.49, *p* < 0.01). This finding was confirmed by the percent contribution of (Bio11) generated by the Jackknife’s test for this month (88.5%) (Table 5). Mean temperature of wettest quarter (Bio8) shared reduced prediction power with Bio11 through the whole season and reached its maximum in December. However, a negative correlation was recorded with the mean temperature of the driest (Bio9) and wettest (Bio8) quarters during July–August and September, respectively.

Precipitation related variables, LULC and elevation showed less influence in predicting *Cx. quinquefasciatus* distributions and VHC ratios*.* Precipitation variables showed negative influence on the distribution of WNV mosquito vector during April–September, especially annual precipitation (Bio12) and precipitation of the driest month (Bio14). However, the seasonal precipitation (Bio15), during April–August, demonstrated a positive correlation with the distribution of *Cx. quinquefasciatus*. The prediction power of LULC was demonstrated during May–August. Unlike the spatial model, both “Other Urban and Built-up Land” (OUBL) and “Residential-Urban” (RU) settings were found to be positively associated with vector-host contact ratios and the onset of mosquito WNV infection rates during June (Table 5, Figure 3). Similarly, elevation shared a subtle positive prediction power during May and June.

### 3.2. Habitat Suitability Modeling of West Nile Virus Vector

#### Spatial and Temporal Analysis

**Figure 4:**
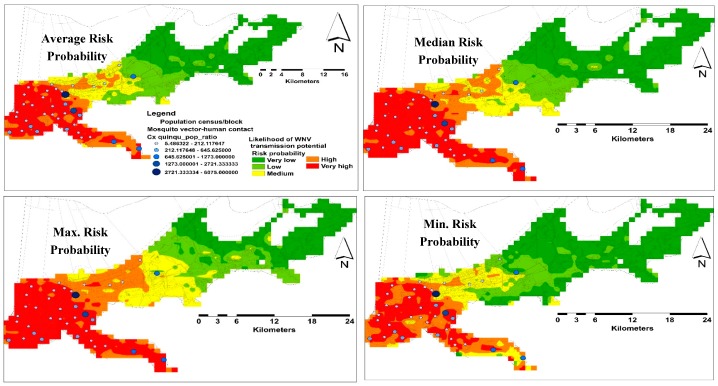
Distribution risk maps for WNV in NOLA representing average, median, maximum, and minimum habitat suitability and sampling localities.

A total of 37 sampling points were included in both spatial and temporal models. Nine and 28 points were used for testing and training the habitat suitability models, respectively. For the spatial model, the average predictive performance was found to be high with an AUC value of 0.85 and 0.71 for training and testing occurrence records respectively, with a standard deviation of 0.07. The specificity of the model was demonstrated as the fractional predicted area. This area at a 10-percentile training presence was found to be 0.36, which were classified as significantly no better than random (*p* < 0.05). The average likelihood of predicting very high risk areas was ~107 km^2^, which is ~9.87% of the total area of the city of New Orleans (Figure 4).

**Figure 5:**
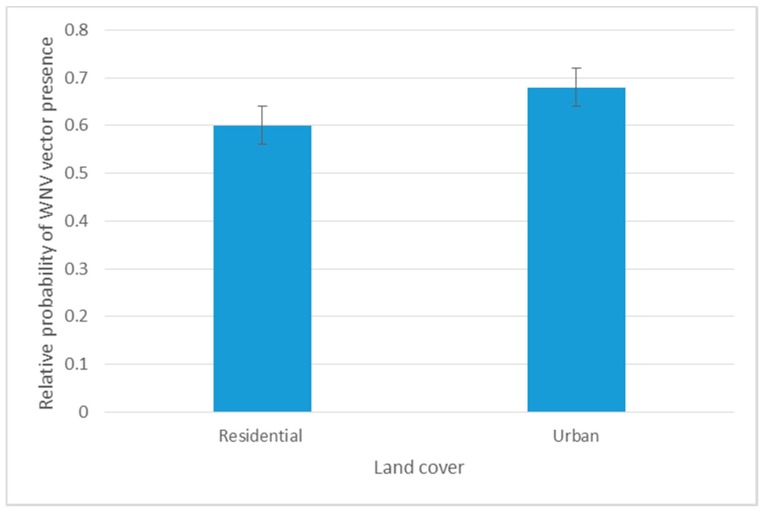
Response curve of spatial distribution of **VHC ratio** to predicting variables in the Jackknife test.

The Jackknife test confirmed the findings we retrieved from RM analysis. The LULC related variables (NFWL, RU, and TD) significantly maximized the predictability of vector-host contact (50.5%). The highest training gain was shared with precipitation related variables (37.4%). Temperature related variables shared a reduced training gain (12.1%) in our model (Table 4). Although RM showed a negative correlation between *Cx. quinquefasciatus* and residential-urban settings, the maximum likelihood of the vector-host contact ratio was predicted at urban areas with less housing structures with an AUC training gain of 0.68 (Figure 5).

For the monthly habitat suitability model, the average predictive performance for the nine months was found to be high with an AUC value of 0.82 and 0.76 for training and testing occurrence records, respectively (Table 3). The fractional predicted area at a 10-percentile training presence was found to be no better than random (*p* < 0.05). The average likelihood of predicting very high suitable habitat was almost ~10.87% of the total area of NOLA. This habitat suitability ranged from ~13.8 and 8.13% during the early and late seasons. The percentage contribution of predicting variables generated by the Jackknife test for each month is summarized in (Table 5).

### 3.3. Model Validation

**Figure 6:**
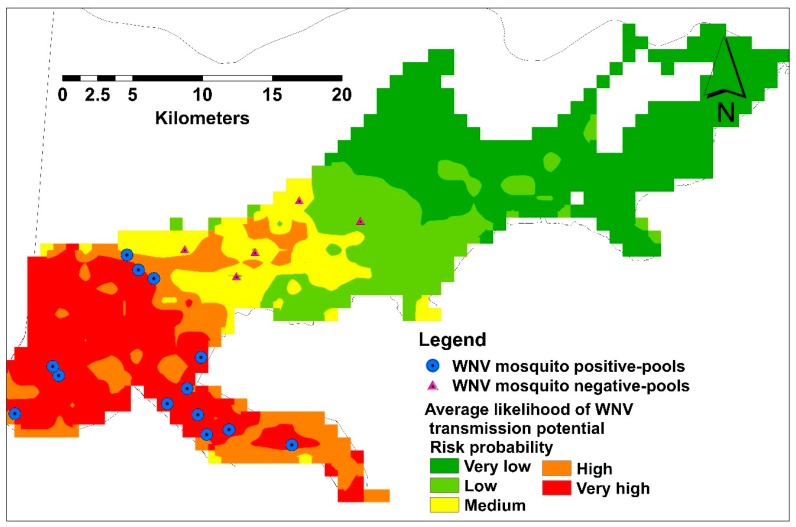
Average habitat suitability of infective mosquito showing field validation points.

A total of 715 mosquito pools were tested for WNV representing 18 collection sites. Only 30 mosquito pools were WNV positive during June–December, confirming our results in the temporal analysis. Thirteen and five collection sites were recorded as WNV positive and negative, respectively, representing different habitat suitability classes (Figure 6).

## 4. Discussion

In our model, we generated habitat suitability estimates for the spatio-temporal likelihood of VHC ratios in NOLA. Since the relationship between mosquito population density and human hosts is important in determining the infective biting rate and transmission risk of arboviruses, density of *Cx. quinquefasciatus* was linked to the human population census in order to generate risk maps for areas under risk of increased VHC. Additionally, we predicted the habitat suitability for the likelihood of VHC within flight ranges of *Cx. quinquefasciatus* (~5-km) around their sampling sites [29,53]. The significant explanatory variables were evaluated and selected using minimum AICc values using stepwise RM. The potentiality of the current prediction model was proven by the high AUC and *R^2^* values produced by MaxEnt and RM. These thresholds indicate that occurrence records were likely assigned a higher probability of presence than background sites. Additionally, the generated risk map was validated using 18 independent field collected sampling points and tested for mosquito WNV infection rates during the season.

The human population in NOLA is centralized in the western areas of the city. However, the likelihood of VHC ratios demonstrated heterogeneous distributions in this side (Figure 4). Although the monthly predicting variables showed some variations, especially the climate, in terms of their percent contribution, the number of these variables declined gradually toward the end of the season. However, the changes in these predicting variables had a consistent influence on the distribution of VHC likelihood of the same high risk areas in the west side of the city. This may reflect the temporal resilience of this mosquito vector to their predicting climate variables in these habitats. This resilience gives the WNV vector the ability to develop and survive in close vicinity to WNV reservoir bird host(s), which was confirmed by the positive WNV mosquito pools during our study [29,53]. However, multi-year mosquito data are recommended to be included in further investigations.

During June and early August 2002, WNV was identified in pools of *Cx*. *quinquefasciatus* mosquitoes in southeastern Louisiana with the possibility of *Cx*. *salinarius* acting as a secondary vector [6]. Although other mosquito vectors were incriminated in amplification and transmission potentials, the selective feeding preference on both human and avian blood and vectorial capacity experiments emphasized that *Cx. quinquefasciatus* is the competent vector in transmitting WNV in LA. The *Cx. quinquefasciatus* mosquito is well known as exophilic and exophagic and the breeding habitats range from ditches, woodland pools, and freshwater marshes of a semi-permanent or permanent nature [25]. As much as *Cx. quinquefasciatus* maintains and amplifies WNV within reservoir host bird(s) [15,16], it is responsible for the urban transmission cycle of WNV in southern and southeastern parts of the USA [17,18,19].

In the spatial analysis, the RM demonstrated a significant association between NFWL and VHC ratios (*R*^2^ = 82, *p* < 0.01). The NFWL habitats were dominant in the eastern side of the city with significantly low human population census. Although this side has not been extensively sampled (No. traps = 1), it is worthy to be highlighted in further investigations to understand the temporal association between WNV vector and reservoir host(s). Other LULC related variables such as TD, OUBL and RU showed a reduced contribution in predicting the likelihood of VHC. These LULC habitats provide both sugar and blood meals, and are favorable to WNV maintenance by enhancing maintenance and amplification phases between mosquito vector and their nesting/roosting reservoir bird hosts, especially the passerines [17]. This finding was confirmed by the selective feeding preference of this mosquito vector in NOLA, their contribution in both enzootic and epidemic transmission cycle of WNV [5,17,54,55,56,57], and the extended transmission season due to the milder climatic conditions of the Gulf Coast as manifested in mosquito WNV infection rates (Figure 3). Similar reports of increases in mosquito WNV infection rates have been made in 2005 and 2006 in Chicago [58]. Moreover, both the Jackknife test in MaxEnt (28.9%) and temporal analysis emphasized the association between LULC types during amplification and early transmission phases (May–August) (Table 3 and Table 5).

Although no WNV human cases were included in our model, the transmission potential still exists due to the high VHC ratios during September and high suitable habitats. In addition, the lack of data on vector competence, survival rate, and the gonotrophic cycle period for NOLA prevented inclusion of this information in our model. However, the high risk probability was generated utilizing WNV minimum infection rates (0.8/1000) [5].

The climate variables shared reduced contributions in predicting likelihood of VHC in the spatial analysis [55,56]. However, climate was demonstrated as the key predicting factor in determining temporal distribution of WNV vectors. The varied correlations between temperature-related variables and VHC explain the association of WNV mosquito distribution with warmer areas during the coldest months. This was confirmed by the negative correlation between VHC and Bio6, which represents the minimum temp of the coldest months. Similarly, Bio6 predicts timing and distribution of the nesting/roosting bird hosts during early spring. In a field study, severe winter caused delay in birds nesting, which explains the variation in timing of host feeding shifts. The VHC showed a negative correlation with precipitation during the driest (Bio17) and warmest (Bio18) quarters [25,55,56]. This negative correlation may be attributed to the flush of limited numbers of mosquitoes from breeding habitats due to rainfall during dry and wet seasons. Subsequently, this reduced the abundance/distribution of WNV mosquito vector during these periods.

In the temporal analysis, we attempted to characterize the ecological resilience of WNV mosquito vector in response to seasonal temperature and precipitation related variables, in association with other LULC variables. This resilience was demonstrated by a decrease in the number of variables that predicted VHC ratios during the late season. The lagged influence of mean temperature of the coldest quarter (Bio11) had a positive association with the increased development/distribution of WNV vector during April–September [25]. This was manifested as maximum prediction probability of VHC ratios during September (*R*^2^ = 58.49, *p* < 0.01). This lagged influence may cause the increase in the development rate of mosquito vectors in affected areas during the amplification phase of the WNV pathogen during April–May. Meanwhile, neotropical bird migrants, mainly passerines, tend to nest during April–May or roost through July, which is crucial for the amplification phase [55,59]. Accordingly, this may accelerate the disease transmission during June–September. Moreover, blood meal preference may shift from birds to mammals including humans that are temporally associated with distribution of nesting/roosting birds, thereby enhancing human risk of arboviruses [55,56,58]. This shift in mosquito feeding preference may be influenced by the temperature-related variables during dry and wet seasons, i.e., negative correlations between VHC and temperature-related variables during dry and wet seasons. The increase in these temperatures reflects the reduction in water bodies that may allow breeding and nesting habitats for mosquito vectors and birds, respectively. Similarly, the negative influence of precipitation variables during April–September, especially annual precipitation (Bio12) and precipitation of the driest month (Bio14), shared a reduced influence toward the prediction of distributions of *Cx. quinquefasciatus.* This may help stimulate blood feeding during these times. The seasonal precipitation (Bio15) during April–August increased both available water habitats and distribution of WNV vector.

Our findings showed heterogeneity in the temporal distribution of *Cx. quinquefasciatus* in response to LULC during May–August. This reflects the influence of some LULC classes on monthly flux and distribution patterns of mosquito populations. The heterogeneity in monthly distributions of mosquito vectors can have a large impact on virus amplification during the early season, especially when it is in close vicinity to reservoir bird hosts. Although no WNV positive mosquito pools were reported during April and May, NFWL was positively correlated with VHC during May (*R*^2^ = 72.6). This may reflect the association between nesting/roosting reservoir bird hosts and VHC ratios in May. During June–August, WNV seemed to build inside mosquito bodies to the detectable level that can cause potential transmission. Both OUBL and RU settings were associated with the onset of mosquito WNV infection rates during June. Other models reported that WNV transmission was either associated with forested and urban land [18,60] or socioeconomic status [61]. However, these correlations may vary spatially or temporally [62].

Some previous studies improved our understanding about biology and ecology of *Cx. quinquefasciatus* and epidemiology [16,21,63]. Nevertheless, their findings did not highlight the interaction between different systems and their overall influence on mosquito vector distribution at a local scale. Moreover, these models predicted the geographic distribution of WNV vectors utilizing mosquito density as sampling points rather than the flight range area and vector-host contact ratios.

Land geomorphology and topography were used in predicting suitable habitats for mosquito breeding [25,34,35,62]. Four potential indicators were rigorously investigated: aspect ratio, slope, land surface curvature and hill shade [25,34,35,62]. The sampled gravid mosquito vectors reflect the proximity of water habitats to collected samples. Although these indicators showed potentiality in predicting WNV mosquito vectors in other areas [25,35], only relative high altitude demonstrated temporal influence on increased VHC ratios during May and June.

## 5. Conclusions

In the current study we modeled the spatio-temporal distribution of VHC ratios in response to future climate scenario, LULC, human population census, and DEM. Vector-host contact (VHC) ratios were estimated as a potential entomological indicator for the likelihood of biting rate and transmission potential of WNV. The VHC ratios were estimated within 5-km buffer zones around mosquito sampling sites representing their average flight range utilizing mosquito density and population census/house block. The likelihood of VHC ratio was first predicted in response to the biophysical systems using stepwise multiple regression model (RM). Accordingly, we used the significant predicting variables from RM to highlight the spatio-temporal distribution of areas under risk of increased VHC emphasizing the likelihood of infective mosquito bites.

The interaction between these different biophysical systems showed heterogeneous influences on the spatio-temporal distribution of VHC ratios. In the spatial analysis, 12 variables were associated with the distribution of VHC ratios, and NFWL showed the highest prediction gain (*R*^2^ = 81.62). Although NFWL has not been sampled extensively during our study (1), to complete our objective, this variable needs to be highlighted rigorously in a separate investigation. Seasonal precipitation- and annual temperature-related variables shared reduced significant associations with VHC ratios. The average likelihood of predicting very high risk areas was ~107 km^2^, which is ~9.87% of the total area of NOLA. Although neither the temporal distribution of VHC ratios nor their estimates were significantly changed from month-to-month (except during September), their monthly response showed resilience to the number and type of the influential factors. The highest VHC ratio was reported during September, which was associated with the peak positive WNV mosquito pools. Seasonal temperature-related variables showed the highest influence on monthly likelihood of VHC in comparison with seasonal precipitation, LULC and DEM variables. This finding was confirmed by both RM (*R*^2^ = 58.49) and Jackknife’s test (88.5%). The influence of LULC on likelihood of VHC was demonstrated during May–August. The NFWL showed a positive association with the increased VHC ratio during May, with no positive WNV mosquito pools. Meanwhile, both OUBL and RU settings were associated with the onset of mosquito WNV infection rates during June. This may reflect the distribution of WNV vector in close vicinity to reservoir host(s) during May, during the virus amplification phase, and the virus beginning to build up inside mosquito bodies during June–August. During September–December, reduced numbers of positive WNV mosquito pools were recorded, which may explain the reduced viremia in wild *Cx. quinquefasciatus*.

The independent field collected sampling points were consistent with both likelihood of VHC ratios and spatio-temporal distribution of increased VHC ratios. However, multi-year mosquito, spatial projections of LULC and human population census data are recommended to be included in further investigations. Moreover, due to data limitation of reservoir host(s), human cases, mosquito vector survival rates, vector capacity parameters, and gonotrophic periods, we did not have the chance to include these variables in our study.

## Figures and Tables

**Table 1:** Proposed thirty-three variables in prediction model of WNV mosquito vectors in the city of New Orleans, LA.

Variable	Variable Name	Data Source	Units
Alt	Elevation in meters	WorldClim ^1^	Meter
Aspect	Aspect ratio	Generated ^2^	Degrees
Bio01	Annual Mean Temperature	WorldClim ^1^	Degree Celsius
Bio02	Mean Diurnal Range (Mean of monthly (max temp − min temp))	WorldClim ^1^	Degree Celsius
Bio03	Isothermality (BIO2/BIO7) (* 100)	WorldClim ^1^	Dimensionless
Bio04	Temperature Seasonality (standard deviation * 100)	WorldClim ^1^	Degree Celsius
Bio05	Max Temperature of Warmest Month	WorldClim ^1^	Degree Celsius
Bio06	Min Temperature of Coldest Month	WorldClim ^1^	Degree Celsius
Bio07	Temperature Annual Range (BIO5-BIO6)	WorldClim ^1^	Degree Celsius
Bio08	Mean Temperature of Wettest Quarter	WorldClim ^1^	Degree Celsius
Bio09	Mean Temperature of Driest Quarter	WorldClim ^1^	Degree Celsius
Bio10	Mean Temperature of Warmest Quarter	WorldClim ^1^	Millimeter
Bio11	Mean Temperature of Coldest Quarter	WorldClim ^1^	Millimeter
Bio12	Annual Precipitation	WorldClim ^1^	Millimeter
Bio13	Precipitation of Wettest Month	WorldClim ^1^	Millimeter
Bio14	Precipitation of Driest Month	WorldClim ^1^	Millimeter
Bio15	Precipitation Seasonality (Coefficient of Variation)	WorldClim ^1^	Fraction
Bio16	Precipitation of Wettest Quarter	WorldClim ^1^	Millimeter
Bio17	Precipitation of Driest Quarter	WorldClim ^1^	Millimeter
Bio18	Precipitation of Warmest Quarter	WorldClim ^1^	Millimeter
Bio19	Precipitation of Coldest Quarter	WorldClim ^1^	Millimeter
Curvature	Curvature	Generated ^2^	Degrees
DFL	Deciduous forest land	USGS ^3^	Integer values
FW	Forested wetland	USGS ^3^	Integer values
Hill shade	Hill shade	Generated ^2^	Degrees
ICS	Industrial and commercial services	USGS ^3^	Integer values
NFWL	Non-forested wetland	USGS ^3^	Integer values
OUBL	Other urban and build-up land	USGS ^3^	Integer values
Population census	Population census per block	NOLA ^5^	No. household/block
RU	Residential and urban settings	USGS ^3^	Integer values
SCLRE	Streams, canals, lakes, reservoirs and estuaries	USGS ^3^	Integer values
Slope	Slope	Generated ^2^	Degrees
TD	Tree density	Lewis et al. ^4^	No. trees/area

^1^ WorldClim Global Climate database v1.4, available at: http://www.ccafs-climate.org/data/ (accessed on 7 March 2016); ^2^ Digital elevation model using the surface spatial analyst tool in Arc tool box of ArcGIS ver. 10.1; ^3^ USGS available at: http://water.usgs.gov/GIS/dsdl/ds240/ (accessed on 3 March 2016); ^4^ Lewis et al. (In Review) [31]; ^5^ data.nola.gov (accessed on 7 March 2016) All layers of variables data used in producing species distribution model gridded to ~1 km spatial resolution and projected into World Geodetic System (WGS) 1984.

**Table 2:** Number of traps and area percentage of LULC classes within 5-km buffer radii in NOLA.

LULC Class	Area % LULC Class	No. Traps/Class
Deciduous forest	7.18	2
Forested wetland	8.01	2
Industrial and commercial services	9.56	4
Non forested wetland	7.72	1
Other urban and built-up land	4.61	3
Residential-Urban	28.29	25
Streams, canals, lakes, reservoirs and estuaries	24.93	0

**Table 3:** Predicting variables used in building up the spatial and temporal models.

Model	Variables	Test AUC	*R*^2^	AICc
Spatial Model	Bio 1, 2, 4, 6, 7, 11, 13, 17, 18, NFWL, RU, TD	0.71	0.82	505.01
April	Bio 11, 12, 14, 15, 8	0.77	0.75	79.05
May	Alt, Bio 8, 11, 12, 14, 15, NFWL	0.74	0.85	71.53
June	Alt, Bio 11, 14, 2, 8, OUBL	0.73	0.81	80.57
July	bio14, 15, 8, 9, OUBL	0.77	0.83	73.28
August	bio11, 12, ,15, 8, 9, OUBL, RU	0.8	0.93	60.67
September	Bio 8, 11, 12	0.79	0.58	88.26
October	Bio 1, 8, 12	0.75	0.55	81.30
November	Bio 2, 8	0.78	0.52	84.34
December	Bio 8, 12	0.71	0.42	80.95

**Table 4:** Percent contribution of predicting variables on spatial distribution of WNV mosquito vector during 2015 in City of New Orleans, LA.

Variable	Linear Regression Analysis				% Contribution (Jackknife’s Test)
	Coefficient	*R*^2^	*p*	AICc	
Bio 1	3.08	0.65	8	635.73 **	1.1
Bio 11	4.74	0.46	4	722.22 **	8.8
Bio 13	0.77	0.79	10	532.48 **	0.1
Bio 17	-0.24	0.08	2	834.61 *	0.1
Bio 18	-0.25	0.73	9	579.02 **	37.2
Bio 2	-0.83	0.81	12	514.95 *	0.2
Bio 4	0.07	0.11	3	828.82 **	1.6
Bio 6	-2.99	0.49	5	711.32 *	0.1
Bio 7	-1.74	0.57	6	676.71 *	0.3
NFWL	1.91	0.82	13	505.01	28.9
RU	-1.24	0.60	7	662.57 **	20.9
TD	0.60	0.80	11	523.11 **	0.7

* Significant at *p* < 0.05; ** Significant at *p* < 0.01; Best predictor, significant (*p* < 0.01).

**Table 5:** Percent contribution of predicting variables on temporal distribution of vector-host contact ratios of WNV in the City of New Orleans, LA.

Month	Linear Regression Model					% Contribution (Jackknife’s Test)
	Variable	Coefficient	*R*^2^	*p*	AICc	
**April**	Bio 11	1.5671996	0.7468	6	79.0541	73.6
	Bio 12	−0.1904792	0.6252	5	83.8586 **	1.7
	Bio 14	0.580292	0.4581	3	85.311 *	0.8
	Bio 15	0.9505776	0.5683	4	83.3859 *	1.9
	Bio 8	1.549815	0.292	2	88.5009 **	21.9
**May**	Alt	0.2054192	0.6929	5	74.2539 *	2.2
	Bio 11	1.2170901	0.8465	8	71.5334	51.1
	Bio 12	−0.1428952	0.7531	7	77.6814 **	1.7
	Bio 14	−0.0756836	0.5338	3	77.4397 *	0.9
	Bio 15	0.8079636	0.6232	4	75.555 **	1.2
	Bio 8	1.9098991	0.3773	2	81.4792 **	15.4
	NFWL	1.6682811	0.726	6	75.5791 **	27.6
**June**	Alt	0.2648213	0.8139	7	80.5656	1.6
	Bio11	1.0789617	0.7693	6	81.1173 **	57.9
	Bio14	−1.0029246	0.5241	3	87.6001 **	1.1
	Bio2	1.4828419	0.5889	4	87.3124 **	0.5
	Bio8	2.3433611	0.2854	2	94.4485 **	38.8
	OUBL	1.5141391	0.6708	5	85.5914 **	0.1
**July**	Bio14	−0.6971647	0.5415	3	86.6717 **	2.1
	Bio15	1.5743201	0.7435	5	79.5723 **	1.5
	Bio 8	2.4390573	0.3209	2	93.1952 **	31
	Bio 9	−0.6412268	0.8334	6	73.2771	65.3
	OUBL	1.9151789	0.6321	4	84.6186 **	0.1
**August**	Bio 11	1.0996856	0.8687	6	65.7127 **	35.1
	Bio12	−0.0854343	0.6302	4	82.893 **	1.2
	Bio15	1.5403677	0.8289	5	68.006 **	0.3
	Bio8	1.8883324	0.1999	2	95.2837 **	9.7
	Bio9	−0.7676054	0.9091	7	61.4863 *	16.1
	OUBL	2.9633858	0.4018	3	91.2107 **	1.3
	RU	0.2968421	0.9294	8	60.6681	36.3
**September**	Bio 8	−0.1073224	0.2915	2	95.6155 **	10.1
	Bio 11	0.9622917	0.5849	4	88.2612	88.5
	Bio 12	−0.1073224	0.4927	3	90.1222 **	1.4
**October**	Bio 1	1.2929084	0.5465	4	81.2964	56.6
	Bio 8	1.431466	0.1947	2	89.6387 **	37.7
	Bio 12	−0.0983597	0.4436	3	83.2546 **	5.7
**November**	Bio 2	0.9214511	0.5155	3	84.3401	0.1
	Bio 8	1.6743075	0.2768	2	91.4944 **	99.9
**December**	Bio 8	1.591142	0.1993	2	85.9884 **	84.9
	Bio 12	−0.0493324	0.4161	3	80.9488	15.1

* Significant at *p* < 0.05; ** Significant at *p* < 0.01; Best predictor, significant (*p* < 0.01).
